# P-51. Influenza Vaccine Effectiveness in Ambulatory Care Settings among Pregnant Persons in the 2023-2024 Influenza Season

**DOI:** 10.1093/ofid/ofae631.258

**Published:** 2025-01-29

**Authors:** Emily L Reeves, Zachary Weber, Duck-Hye Yang, Sarah W Ball, Sara Y Tartof, Lina S Sy, Suzanne B Salas, Cassandra Bezi, Bruno Lewin, Kristin K Dascomb, Julie Arndorfer, Daniel Bride, Nicola P Klein, Kristin Goddard, Bruce Fireman, Ousseny Zerbo, John R Hansen, Julius Timbol, Lawrence Block, Toan Ong, Michelle Barron, David Mayer, Malini DaSilva, Gabriela Vazquez-Benitez, Allison L Naleway, Stephanie Irving, Ruth Link-Gelles, Samantha M Olson, Katherine Adams, Varsha Neelam, Sascha Ellington, Shikha Garg, Mark W Tenforde

**Affiliations:** CDC, Atlanta, Georgia; Westat, Rockville, Maryland; Westat, Rockville, Maryland; Westat, Rockville, Maryland; Kaiser Permanente Southern California, Pasadena, CA; Kaiser Permanente Southern California, Pasadena, CA; Kaiser Permanente Southern California, Pasadena, CA; Kaiser Permanente Southern California, Pasadena, CA; Kaiser Permanente Department of Research and Evaluation, Pasadena, CA; Intermountain Healthcare, Murray, Utah; Intermountain Healthcare, Murray, Utah; Intermountain Healthcare, Murray, Utah; Division of Research Kaiser Permanente Vaccine Study Center, Oakland, California; Division of Research Kaiser Permanente Vaccine Study Center, Oakland, California; Division of Research Kaiser Permanente Vaccine Study Center, Oakland, California; Division of Research Kaiser Permanente Vaccine Study Center, Oakland, California; Division of Research Kaiser Permanente Vaccine Study Center, Oakland, California; Kaiser Permanente Vaccine Study Center, Oakland, CA; Kaiser Permanente Northern California, Oakland, California; University of Colorado Anschutz Medical Campus, Centennial, Colorado; University of Colorado, Aurora, Colorado; University of Colorado Anschutz Medical Campus, Centennial, Colorado; HealthPartners Institute, Minneapolis, MN; HealthPartners Institute, Minneapolis, MN; Kaiser Permanente Center for Health Research, Portland, Oregon; Kaiser Permanente Center for Health Research, Portland, Oregon; Centers for Disease Control and Prevention, Atlanta, Georgia; Centers for Disease Control and Prevention, Atlanta, Georgia; CDC, Atlanta, Georgia; CDC, Atlanta, Georgia; CDC, Atlanta, Georgia; Centers for Disease Control and Prevention, Atlanta, Georgia; US Centers for Disease Control and Prevention, Decatur, Georgia

## Abstract

**Background:**

Despite increased risk of influenza-associated complications in pregnant people, influenza vaccination coverage has decreased in recent seasons as vaccine confidence has declined. The purpose of this study was to evaluate influenza vaccine effectiveness (VE) against influenza-associated ambulatory encounters among pregnant persons during the 2023-2024 season, and to compare VE in pregnant versus non-pregnant persons.

**Methods:**

During October 15, 2023 – April 10, 2024, a test-negative, case-control analysis was conducted among pregnant and non-pregnant persons aged 18-49 years with documented female sex. Persons with acute respiratory illness (ARI)-associated emergency department or urgent care visits and who underwent molecular assay testing for influenza at six health systems in the VISION Network were included. VE was estimated using logistic regression comparing the odds of influenza vaccination (≥14 days before the encounter) in influenza positive cases vs influenza negative controls, adjusting for site, age, race/ethnicity, calendar time, and gestational age at encounter (where applicable).

**Results:**

Among 2,439 ARI encounters in pregnant people, 773 (32%) tested positive and 1,666 (68%) tested negative for influenza; 205 (27%) cases vs 608 (36%) controls were vaccinated. Median gestational age at encounter was 20 weeks (IQR: 11-29). Among 40,374 ARI encounters in non-pregnant people, 10,599 (26%) tested positive and 29,775 (74%) tested negative for influenza; 1,727 (16%) cases vs 8,117 (27%) controls were vaccinated. VE was 46% (95% CI: 33-56) in pregnant people and 53% (95% CI: 50-56) in non-pregnant persons.

**Conclusion:**

Influenza vaccination during the 2023-2024 season was effective against influenza-associated ambulatory encounters in pregnant people. VE was similar in pregnant and non-pregnant persons of reproductive age. Vaccination was effective in reducing influenza-associated ambulatory encounters by approximately 50%, yet less than half of the population included in this analysis received the current influenza vaccine. Demonstrating that vaccines are effective can help increase vaccine confidence and may lead to increased uptake.

**Disclosures:**

**Sara Y. Tartof, PhD MPH**, GSK: Grant/Research Support|Pfizer Inc: Grant/Research Support **Lina S. Sy, MPH**, Dynavax: Grant/Research Support|GlaxoSmithKline: Grant/Research Support|Moderna: Grant/Research Support **Nicola P. Klein, MD, PhD**, CSL Seqirus: Grant/Research Support|GlaxoSmithKline: Grant/Research Support|Merck: Grant/Research Support|Pfizer: Grant/Research Support|Sanofi Pasteur: Grant/Research Support **Toan Ong, PhD**, Patent Title: Systems and Methods For Record Linkage: PCT/US2018/047961 Patent Title: Systems and Methods For Record Linkage|PCORI: Travel support to attend the PCORI Annual meeting in Washington DC, 2023|Regenstrief Institute: Advisor/Consultant|Regenstrief Institute: Travel support to attend the OHIE 23 meeting in Malawi. **Michelle Barron, MD**, Innoviva Specialty Therapeutics: Speaker bureau participant **Malini DaSilva, MD, MPH**, CDC, Vaccine Safety Datalink, Center for Excellence in Newcomer Health: Grant/Research Support **Gabriela Vazquez-Benitez, PhD, MSc**, Abbvie: Grant/Research Support|Sanofi Pasteur: Grant/Research Support

